# Structure, toxicity and antibiotic activity of gramicidin S and derivatives

**DOI:** 10.1007/s10096-016-2595-y

**Published:** 2016-02-17

**Authors:** J. Swierstra, V. Kapoerchan, A. Knijnenburg, A. van Belkum, M. Overhand

**Affiliations:** Department of Medical Microbiology and Infectious Diseases, Erasmus University Medical Centre, ‘s Gravendijkwal 230, 3015 CE Rotterdam, The Netherlands; Faculty of Science, Bio-Organic Synthesis, Gorlaeus Laboratories, Leiden Institute of Chemistry, Einsteinweg 55, 2333 CC Leiden, The Netherlands; bioMérieux, R&D Microbiology, 3 Route de Port Michaud, La Balme-Les-Grottes, 38390 France

## Abstract

Development of new antibiotics is declining whereas antibiotic resistance is rising, heralding a post-antibiotic era. Antimicrobial peptides such as gramicidin S (GS), exclusively topically used due to its hemolytic side-effect, could still be interesting as therapeutic compounds. By modifying the amino-acid composition of GS, we synthesized GS analogues. We now show that derivative VK7 has a lower MIC (7.8–31.2 μg/ml, median 15.6 μg/ml) against strains of multi-drug resistant (MDR) *Klebsiella pneumoniae, Acinetobacter baumannii*, and *Pseudomonas aeruginosa* than GS has (3.9–62.5 μg/ml, median 31.3 μg/ml). Low MICs for both VK7 and GS were observed for *Staphylococcus aureus* and *Enterococcus faecium*. VK7 showed reduced haemolysis and less lactate dehydrogenase release. All compounds were fully bactericidal at MIC values. Modification of GS enables production of novel derivatives potentially useful for systemic treatment of human infections.

## Introduction

Six bacterial pathogens with a propensity for developing multi-drug resistance (MDR) are specifically warned for by the Infectious Disease Society of America (IDSA) (ESKAPE: *Enterococcus faecium*, *Staphylococcus aureus, Klebsiella pneumoniae, Acinetobacter baumannii, Pseudomonas aeruginosa*, and extended spectrum beta lactamase (ESBL)-producing Enterobacteriaceae). These species are causing the majority of human infections and efficiently acquire additional resistance traits [1], which implies that new antibiotics have to be effective against these actively evolving MDR pathogens. The incidence of vancomycin-resistant Enterococci (VREs) has increased dramatically over recent years [2]. *S. aureus*, especially methicillin-resistant *S. aureus* (MRSA), currently causes more deaths in the USA annually than HIV and tuberculosis combined [2]. ESBLs continue to be on the rise and limit treatment options [3]. *P. aeruginosa* is becoming increasingly resistant to multiple classes of drugs [4], whereas *Acinetobacters* are naturally resistant to many classes of antibiotics [5]. Increasing antibiotic resistance leads to extended hospitalization, rising treatment costs, and increased morbidity and mortality.

The rise of antibiotic-resistant pathogens has sparked research into currently disregarded antimicrobial peptides including gramicidin S (GS). GS is naturally produced by *Aneurinibacillus migulanus* [6] and was first discovered in 1941 [7]. GS shows antimicrobial activity against both Gram-positives and Gram-negatives in a MIC range from 4–64 μg/ml [8]. The lowest MICs are seen for Gram-positive bacterial species [9]. Despite its good antimicrobial activity, GS cannot be used systemically due to its haemolytic side-effect [10] and is therefore only applied topically to treat superficial infections [11]. GS is a cyclic, C2-symmetrical decapeptide with the sequence cyclo(Pro-^D^Phe-Leu-Orn-Val)_2_. The two Pro-^D^Phe dipeptides form two type II β-turns, and the two Leu-Orn-Val stretches form an antiparallel β-sheet. GS has been reported to kill bacteria by forming pores in the outer membranes [8]. Native GS is a natural scaffold for amino acid alteration in such a way that antimicrobial activity is retained but toxicity is reduced. In previous attempts to modify GS, several strategies have been followed: non-natural amino-acids were included [12], the size of the ring has been modified [8], and the β-turn region [13] and β-strand region have been changed [14]. Still, few new derivatives of GS have been identified that show retention of antimicrobial activity with reduced toxicity [8, 15–18]. We here study the β-strand-modified GS analogue VK7 [14] and the β-turn modified derivative 20 [13]. Studying naturally occurring antimicrobial peptides such as GS could help with the design and development of novel derivative drugs to combat multidrug resistance.

## Material and methods

### ESKAPE panel collection and characterization

Except for the *S. aureus* USA 300 and MRSA 252 strains [19], ESKAPE strains were collected at the Department of Medical Microbiology and Infectious Disease in the Erasmus Medical Centre, Rotterdam, The Netherlands. Thirty clinical isolates of *E. faecium* (5)*, S. aureus* (5)*, K. pneumonia* (5)*, A. baumannii* (5)*, P. aeruginosa* (5) and *E. cloacae* (5) were isolated from January 2010 to October 2011 from different wards (Table 1). All strains were cultured on Columbia agar plates with 5 % sheep blood (Becton Dickinson, Breda, The Netherlands) overnight at 37 °C before antibiotic susceptibility testing. Antibiotic resistance was determined using disk diffusion following Clinical and Laboratory Standards Institute (CLSI) guidelines and VITEK2 (bioMérieux, Zaltbommel, The Netherlands) following manufacturer’s protocol; susceptibility was determined using EUCAST breakpoints [20].

**Table 1:** Review of clinical isolates, their MICs to GS and derivatives, toxicity levels, and therapeutic indices

	Strain #	3	20	GS	VK-7
*Enterococcus faecium*	1	7.8	15.6	3.9	3.9
	2	7.8	15.6	3.9	3.9
	3	7.8	7.8	3.9	3.9
	4	7.8	7.8	3.9	3.9
	5	7.8	3.9	3.9	3.9
*Staphylococcus aureus*	1	31.3	7.8	3.9	7.8
	2	7.8	3.9	3.9	3.9
	3	62.5	62.5	7.8	15.6
	4	31.3	7.8	3.9	7.8
	5	31.2	7.8	3.9	7.8
*Klebsiella pneumoniae*	1	62.5	62.5	31.3	15.6
	2	31.3	62.5	62.5	15.6
	3	62.5	62.5	62.5	15.6
	4	31.3	62.5	31.3	15.6
	5	15.6	15.6	7.8	15.6
*Acinetobacter baumannii*	1	31.3	62.5	31.3	15.6
	2	31.3	62.5	62.5	7.8
	3	62.5	62.5	31.3	15.6
	4	62.5	62.5	15.6	15.6
	5	31.3	62.5	62.5	31.2
*Pseudomonas aeruginosa*	1	31.3	62.5	31.3	7.8
	2	31.3	31.3	31.3	7.8
	3	31.3	31.3	31.3	7.8
	4	62.5	62.5	62.5	7.8
	5	62.5	62.5	62.5	7.8
*Enterobacter cloacae*	1	3.9	7.8	3.9	7.8
	2	3.9	7.8	7.8	15.6
	3	7.8	7.8	3.9	7.8
	4	3.9	1.95	1.95	7.8
	5	62.5	62.5	62.5	15.6
Toxic dose 50 % (hemolyse)		41.6	nd	35.2	nd
Toxic dose 50 % (LDH)		49.8	62.5	18.7	nd
MIC lowest values		3.9	1.95	1.95	3.9
MIC highest values		62.5	62.5	62.5	31.2
TI following hemolysis range		0.66–10.67	nd	0.56–18.05	nd
TI following LDH release range		0.80–12.77	1–32.05	0.30–9.9	nd

Note: nd: not determined since the maximum concentration did not cause toxicity.

### MIC determination

**Fig. 1:**
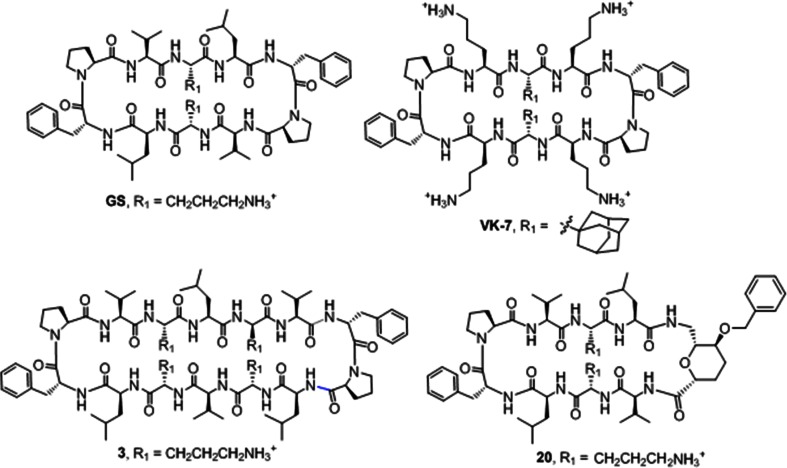
Structure of GS and the three derivatives studied here

Antimicrobial activity of GS and derivatives (Fig. 1) was determined following the CLSI guidelines and in triplicate [21]. Briefly, bacterial cells were cultured overnight on Columbia agar plates with 5 % sheep blood (Becton Dickinson, Breda, The Netherlands). Colonies were suspended in 0.9 % NaCl to a density of 0.5 McFarland, then diluted 1:100 in Müller–Hinton Broth (MHB, Oxoid, Badhoevedorp, The Netherlands); 100 μl of this suspension was added to wells containing GS, derivatives 3, 20, or VK7 ranging from a concentration of 0.95 μg/ml to 62.5 μg/ml in MHB. Ninety-six well plates (Greiner Bio One, Alphen aan den Rijn, The Netherlands) were incubated for 18–24 hours at 37 °C, and MIC values were determined visually. To determine whether antimicrobial activity was bactericidal, 200 μl of the suspension was plated onto new Columbia agar plates with 5 % sheep blood, and colonies were counted.

### Haemolysis assay

Haemolysis assays were performed as described before [13]. Freshly drawn heparinised blood from healthy volunteers was centrifuged for 10 min at 1000 g at 10 °C. The pellet was washed three times with 0.9 % saline and diluted with saline to a 1/25 packed volume of red blood cells. Triton-X100 (1 %) was used as a positive control. GS or derivative 3, 20, and VK7 were diluted in 100 μl PBS in U-bottom 96-well plates (Greiner Bio One). Serial dilution resulted in a concentrations ranging from 62.5 μg/ml to 0.95 μg/ml. DMSO (Sigma–Aldrich, Zwijndrecht, The Netherlands) was used as a solvent control. Subsequently, 50 μl of the red blood cell suspension was added to the wells, and the plates were incubated at 37 °C for 4 h. After incubation, the plates were centrifuged at 1,000 g at 10 °C for 4 min, and 50 μl of the supernatant of each well was dispensed into new flat-bottom 96-well plates (Greiner Bio One), and absorbance was measured at 415 nm in a Bio-Rad 680 spectrophotometer (Bio-Rad, Veenendaal, The Netherlands). OD values were plotted as a percentage of the positive control. Experiments were performed in triplicate.

### Cytotoxicity testing

Human colorectal adenocarcinoma cells (HT-29, ATCC number HTB-38, Wesel, Germany) were cultured in Dulbecco’s Modified Eagle’s Medium (DMEM) (Gibco, Bleiswijk, The Netherlands) with 10 % FCS (Gibco) and penicillin–streptomycin (Gibco). Colourless DMEM (Gibco) with 1 % FCS (Gibco) was used as assay medium. HT-29 cells were seeded at a density of 2.0 × 10^4^ cells/well in a Costar flat-bottom 96-well plate (Corning, Amsterdam, The Netherlands) and incubated overnight. Serial dilutions of GS and derivatives were added and incubated for 4 h at 37 °C. Plates were centrifuged for 10 min at 1,000 g, and the amount of LDH in the supernatants was determined (LDH release kit, Roche, Woerden, The Netherlands) following the protocol. Cytotoxicity testing was independently repeated three times.

### Therapeutic indices

The therapeutic index was defined as a measure of toxicity, either the 50 % haemolysis or the 50 % LDH release, divided by the lowest and highest MIC values seen for each strain tested. Therapeutic indices are given as a range to be compared between GS and its GS derivatives to determine improved performance.

## Results

### Cohort collection and characterization

Five strains each of *E. faecium, S. aureus, K. pneumoniae, A. baumannii, P. aeruginosa*, and *E. cloacae* were collected from various wards of the Erasmus MC in 2010 and 2011 (Table 1). Extensive antibiotic resistance was observed. *S. aureus* and *E. faecium* showed resistances to penicillins, cephalosporins, lincosamides, tetracyclines, macrolides, fusidic acid, aminoglycosides, carbapenems, (fluoro)quinolones, oxazolidonones, monoxycarbolic acid, nitrofuran derivatives, rifamycins, sulfanomides, and glycopeptides. Extensive drug resistance was also observed among *K. pneumoniae, A. baumannii, P. aeruginosa*, and *E. cloacae* including aminoglycosides, (ureido)penicillins (in combination with beta lactamase inhibitors), fluoroquinolones, polymyxins, carbapenems, nitrofuran derivatives, and trimethroprim with sulfamethoxazole. Each strain showed a unique profile with resistance to several clinically used antibiotics.

### MIC determination

**Fig. 2:**
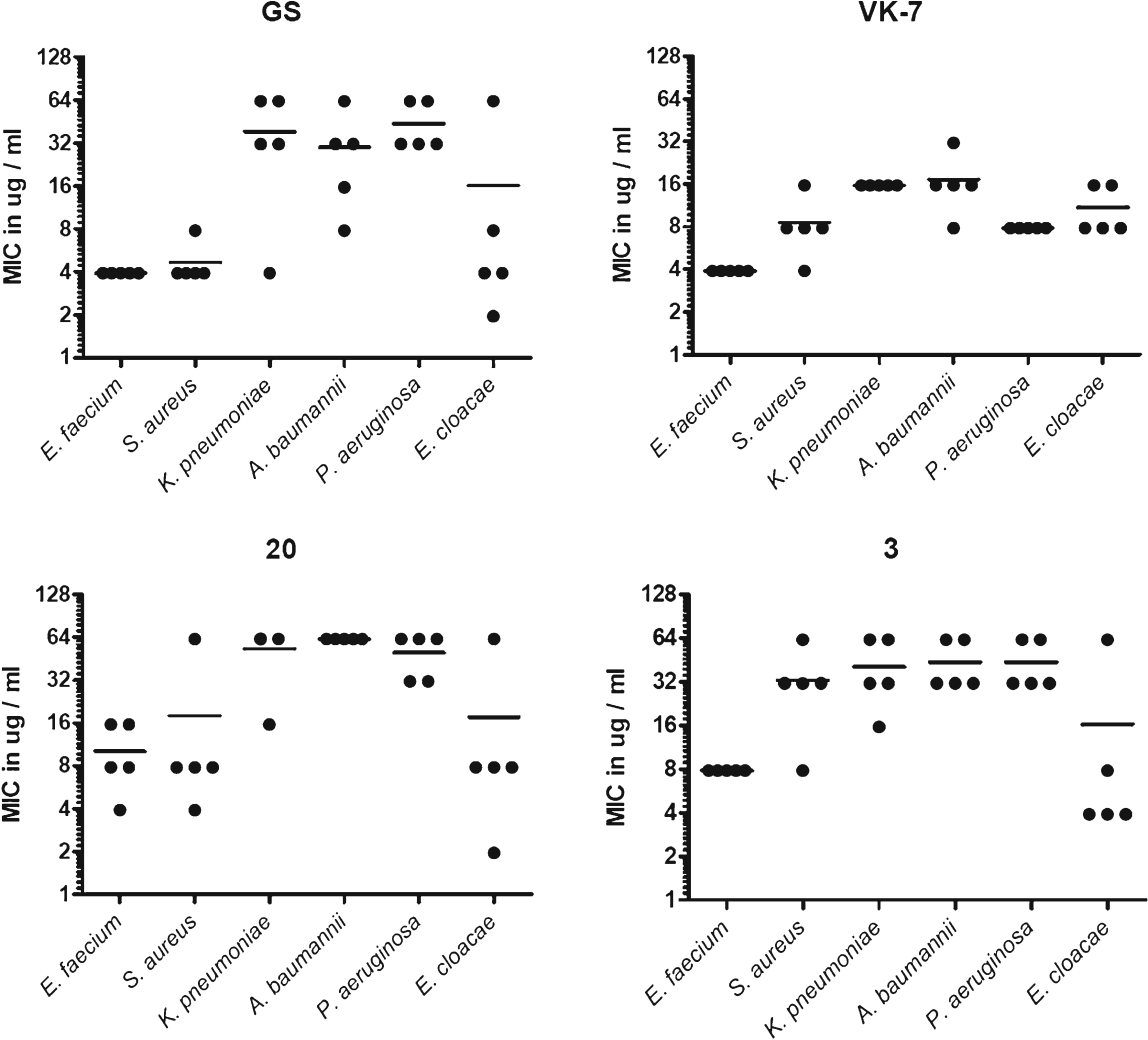
MIC values for GS and its derivatives as defined for a collection 30 ESKAPE strains

GS was active against *S. aureus* and *E. faecium* at 3.9–7.8 μg/ml (median 3.9 μg/ml) (Fig. 2). MIC values against *E. cloacae*, *P. aeruginosa, K. pneumoniae* and *A. baumannii* for GS ranged between 3.9–62.5 μg/ml (median 31.3 μg/ml).

The beta-strand variant VK7 showed activity against *S. aureus* and *E. faecium* in the range of 3.9–15.6 μg/ml (median 3.9 μg/ml), comparable to parental GS. VK7 showed activity in the range of 7.8–31.2 μg/ml (median 15.6 μg/ml) against *E. cloacae*, *P. aeruginosa, K. pneumoniae*, and *A. baumanii*. The MIC values against all *P. aeruginosa* and *A. baumannii* strains and most *K. pneumoniae* strains are 2- to 8-fold lower for VK7 than the GS MIC values.

The β-turn variant 20 showed slightly reduced activity against *S. aureus* and *E. faecium* as compared to GS. MIC values for compound 20 were in the range of 7.8–62.5 μg/ml (median 7.8 μg/ml). Derivative 20 showed activity against *E. cloacae*, *P. aeruginosa, K. pneumoniae*,and *A. baumannii* in the range of 1.95–62.5 μg/ml (median 62.5 μg/ml), which is slightly less than measured for the parental compound.

Derivative 3 showed activity against *S. aureus* and *E. faecium* in the range from 7.8–62.5 μg/ml (median 7.8 μg/ml), which is slightly less than parental GS. Derivative 3 shows activity against the MDR Gram-negative strains *E. cloacae*, *P. aeruginosa, K. pneumoniae*, and *A. baumannii* in the range from 3.9–62.5 μg/ml (median 31.3 μg/ml), which is comparable to the parental compound.

All compounds tested were bactericidal, as sub-culturing of medium from wells without visible growth on agar media did not result in detectable growth.

### Haemolysis assay and therapeutic indices

**Fig. 3:**
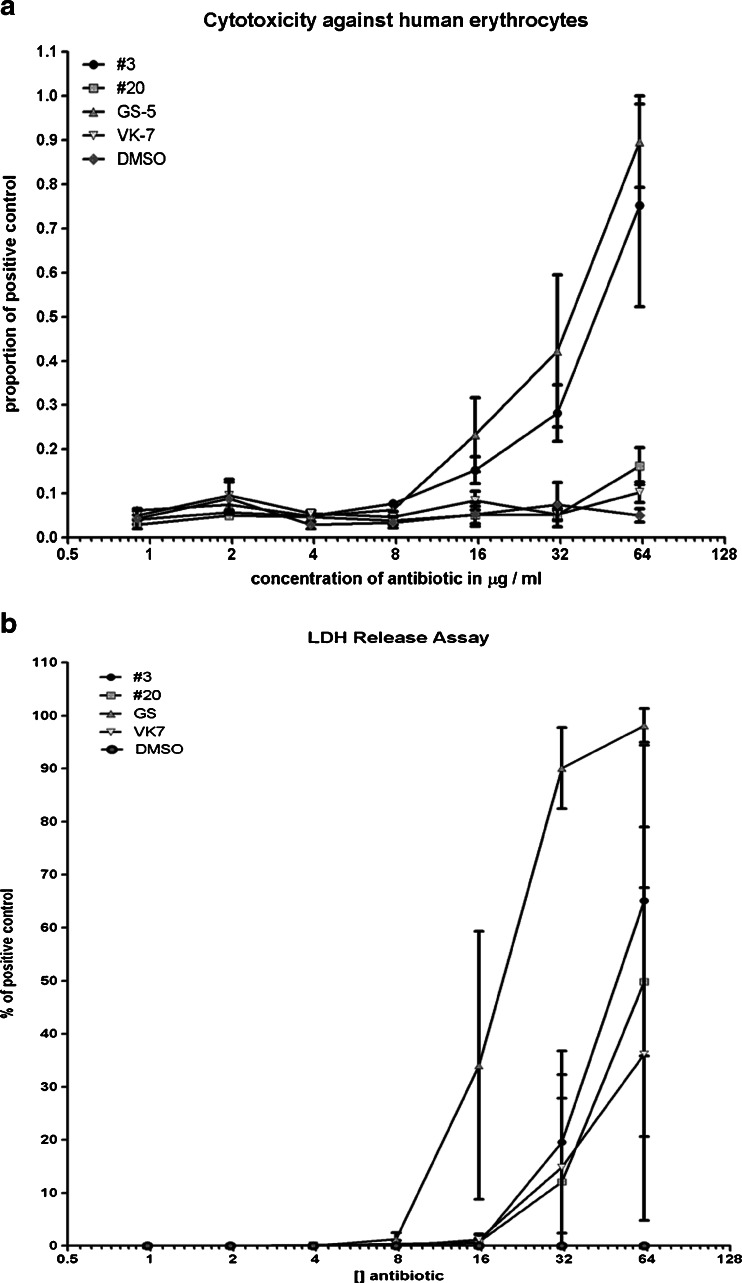
Erythrocyte lysis and LDH release under the influence of GS and the derivatives

Haemolysis is clearly concentration-dependant. Canonical GS showed 50 % haemolysis at 35.2 μg/ml (Fig. 3a). As the MIC values of GS varied from 3.9 to 62.5 μg/ml, the TI_hae_ for GS was calculated to be between 0.56 and 18.5. Derivative 3 showed 50 % haemolysis at 41.6 μg/ml (Fig. 3a), which is in a similar range as for the parental compound. As derivative 3 had MIC values varying from 3.9 and 62.5 μg/ml, the TI_hae_ of derivative 3 was calculated to be 0.6 to 10.6, also comparable to the parental compound. This shows that derivative 3 is not an improved antibiotic in comparison with GS. VK7 and compound 20 did not show haemolysis at 62.5 μg/ml, which was the highest concentration tested. Hence, the exact TI_hae_ for compound 20 and VK7 could not be determined, but still, these derivatives are clearly less haemolytic than GS (Fig. 2).

### LDH release assay and therapeutic indices

The values of 50 % LDH release were 18.7 μg/ml for GS (Fig. 3b). As the MIC values were between 3.9 and 62.5 μg/ml, TI_LDH_ was calculated to be between 0.3 and 9.6. Derivative 3 showed 50 % LDH release at 49.8 μg/ml. As the MIC values were between 3.9 and 62.5 μg/ml, TI_LDH_ was calculated to be between 0.8 and 12.8, in the same range as documented for GS. Derivate 20 showed 50 % LDH release at 62.5 μg/ml. As MIC values were between 1.95 and 62.5 μg/ml, TI_LDH_ was calculated to be between 1 and 32.1 μg/ml, which shows slight improvement compared to GS. VK7 did not reach 50 % LDH release at concentrations tested here. Therefore, no TI_LDH_ could be calculated, indicating again that VK7 is less cytotoxic than GS (Table 1).

## Discussion

Beta-strand modification of GS seemed to be promising for the development of new systemically applicable antibiotics. Derivative VK7 showed activity against *E. cloacae* which was equal to that of GS. Against all *P. aeruginosa* and *K. pneumoniae* and most *A. baumannii* there was a 2–8-fold increase in activity. The antimicrobial activity of VK7 against Gram-positive MDR pathogens such as *S. aureus* and *E. faecium* was similar to that of GS. In addition, we observed reduced toxicity for VK7 towards human erythrocytes and the human colorectal adenocarcinoma cell-line HT-29. The β-strand-modified VK7 has the same overall secondary structure as GS, but probably displays an elevated cationic character counterbalanced by two robust hydrophobic adamantane groups. The data indicate that β-strand modification of GS can generate interesting new antibiotics combining anti-microbial activity and lowered toxicity.

The β-turn-modified derivative 20 showed reduced toxicity compared to the parental compound, with a slight decrease in antibiotic activity compared to GS, especially when used against Gram-negatives. This β-turn-modified derivative encompasses a substituted sugar amino acid (SAA) dipeptide isoster as turn mimetic. The six-ring SAA in our lead was found to have better conformational and hydrophobic characteristics than a 4-ring (oxetane) and 5-ring (furanoid) SAA [9]. Beta-turn modification could still be promising, as at least some reduction of toxic potential is observed. Not all modifications of GS have a positive effect on antimicrobial activity and toxicity: derivative 3 shows comparable antimicrobial activity to the parental compound but a similar toxicity profile.

The synthesis of modified GS derivatives has been reported by other groups [8, 12, 15, 17]. One of the key factors important in the balance between cytotoxicity and antimicrobial activity is overall hydrophobicity–hydrophilicity. Derivatives that are slightly less hydrophobic than the parental GS generally show good antimicrobial activity, while showing reduced haemolysis. Using solid- and liquid-phase organic synthesis, derivatives of GS can be obtained containing non-naturally occurring amino acids, which show reduced cytotoxicity and reduced haemolysis, while retaining antimicrobial activity.

Antimicrobial peptides may have a bright future in combating infection, as they generally do not have a single conserved target, but affect multiple bacterial processes. Modifications of the β-strand of GS in which the hydrophobic side chains have been varied are promising leads for the development of novel compounds. New derivatives of GS can possibly address the growing problem of multi-drug resistant bacteria and lead to new therapeutic compounds for systemic use, as is suggested on the basis of our current data.
